# Challenges in organizing quality diabetes care for the urban poor: a local health system perspective

**DOI:** 10.1186/1753-6561-6-S5-O13

**Published:** 2012-09-28

**Authors:** Upendra Bhojani, BS Thriveni, Roopa Devadasan, CM Munegowda, Amruthavalli, Narayanan Devadasan, Bart Criel, Patrick Kolsteren

**Affiliations:** 1 Institute of Public Health, Bangalore, India; 2 Department of Public Health, Institute of Tropical Medicine, Antwerp, Belgium

## Introduction

India is urbanizing at a rapid pace. Moreover, a quarter of the urban population lives in slum areas [1]. Unfavorable social determinants in health and huge inequities in access to healthcare within urban India leave the urban poor with dismal health indicators [2]. The burden from chronic diseases is also on rise in India, disproportionately so for urban population, and is now the leading cause of deaths [3,4]. India is leading the diabetes epidemic in the world [5]. In urban south India, diabetes prevalence is on a rapid rise (from 5% in 1984 to 13.9% in 2000) [6].

There has been a growing concern among public health researchers/programmers regarding the neglect of urban poor in governments’ health policies/programs [7,8]. The government health services remain primarily oriented towards management of acute episodes [9]. In this study, we analyze a local health system in Bangalore’s KG Halli neighborhood, identify the main challenges in organizing the quality diabetes care, and discuss the way forward. KG Halli has a population of over 44,500 with one notified slum area. The median per-capita income is INR 2200/month.

## Methods

KG Halli is the field site of the Urban Health Action Research Project (UHARP) designed and implemented by the Institute of Public Health (IPH). Its purpose is to enhance access to quality healthcare for the KG Halli residents.

We used the data collected over a period of almost four years (2009-2012) through following tools:

(1) A census covering 9,299 households with a response rate of 98.5% in KG Halli using a structured questionnaire collecting data about socio-demographic factors, self-reported illnesses, healthcare seeking behavior and expenditure;

(2) Audio recordings of six of the periodic meetings of healthcare providers in KG Halli, facilitated under the UHARP;

(3) Field notes from UHARP researchers; and

(4) In-depth interviews with eight diabetes patients (sampled purposively to capture diverse experiences of healthcare seeking and living with diabetes), and semi-structured interviews with 14 healthcare providers, staff from two pharmacies and two laboratories in KG Halli (to understand organization of diabetes care, challenges and suggestions for improving diabetes care).

For the survey, interviews, and meetings, an informed consent was obtained prior to collecting data. We used the health system dynamics framework developed by Van Olmen et al [10] as analytical framework to structure our findings. Quantitative data were analyzed using STATA while thematic analysis was done for qualitative data.

## Results

### Mixed healthcare provision

Of the two government facilities in the area, the Community Health Center (CHC), run by the state government, provides care for diabetes. In the private sector, there are at least 18 doctor clinics, four hospitals, three laboratories, and many pharmacies. These clinics are staffed by general practitioners (GPs) who reported their training being in various medical systems; modern medicine (four), unani (eight), ayurveda (four), homeopathy (one) and others.

### Stewardship/regulation

There is a lack of administrative and operational integration across providers. Only two of the private providers were aware of and registered under the Karnataka Private Medical Establishment Act (KPMEA), a mandatory mechanism to ensure minimum quality in private healthcare provision. All GPs, except for three who were not trained in modern medicine, were practicing modern medicine. None of the providers interviewed were aware of the standard treatment guidelines for diabetes developed at national level. Both interviewees who were managing private pharmacies were untrained pharmacists. Pharmacies and laboratories interviewed reported the practice of kickbacks (10 to 25% of investigation or medication costs) given by them (and other laboratories/pharmacies) to most GPs in the area.

### Human-resource/technology/infrastructure

Medical specialists were available with prior appointment in hospitals. The CHC, which is supposed to provide specialist care, neither had specialist services nor offered laboratory investigations for diabetes. Other factors affecting diabetes care at CHC included the frequent stock-outs of diabetes medicines and the limited availability of medical doctors due to frequent deputations and/or turnover. For primary care on an outpatient basis, only five GPs followed some form of medical record system aimed at organizing clinical information over the time. Most GPs prescribed branded medicines and reported negative perceptions of generic medicines (‘not safe’, ‘not as effective as branded medicines’). Though generic medicines were available at some of the private pharmacies, the cost was comparable to that of branded medicines.

### Finances

Over 85% of diabetes patients sought care form private providers that operate on fee-for-service basis. 72% of diabetes patients incurred out-of-pocket (OOP) expenditure for outpatient care and of those 22% spent over 10% of their family income on diabetes care. In 3.3% of the episodes, families resorted to borrowing money and/or selling assets.

People’s participation in the health system was limited to exercising choice in selecting providers, self-care/medication, and funding the system. Public and private orientation to healthcare delivery coexisted with dominance of the latter one.

## Discussion

Some of the challenges highlighted in this paper (e.g., availability of human resource/medicines/laboratory within government sector) are not unique to KG Halli, but affects the government system as a whole. The National Rural Health Mission launched in 2005, aims to address these challenges. Unfortunately, the National Urban Health Mission, an initiative similar to its rural counterpart for urban areas, has remained in draft stage since 2010.

Considering the dominant presence and utilization by diabetes patients of poorly regulated private providers, there is need to enhance the implementation of the existing regulatory mechanisms e.g. KPMAE. In a pluralistic medical system like the one in KG Halli, there is need for some sort of steering mechanism that enhances coordination across health providers and directs the system towards a common goal. The UHARP has been facilitating actions in this direction with some positive outputs including enhanced coordination and collaboration across the providers in addressing local health issues.

High OOP spending and related impoverishment implies the need for financial protection mechanisms. Expanding the coverage of existing schemes (like *Rastriya Swasthya Bima Yojana*) to include urban poor, as well as the service package to include outpatient care for chronic conditions might significantly reduce OOP payments. Motivating providers to prescribe generic medicines through various mechanisms (e.g. incentivizing, changing the perceptions about generic medicines through effective knowledge dissemination) and making generic medicines available at low prices are other ways of reducing OOP spending. Karnataka government has recently implemented a pilot making generic medicines available at low prices at selected tertiary government hospitals. Expansion of such schemes and making outlets within the community would be a positive step.

## Competing interests

The first five authors are part of the team implementing the Urban Health Action Research Project in KG Halli, Bangalore.

## Funding statement

The UHARP is funded by the Sir Dorabaji Tata Trust, MISEREOR, and by the Directorate-General for Development Cooperation and Humanitarian Aid (DGD), Government of Belgium, through the institutional collaboration between the Institute of Public Health, Bangalore and the Institute of Tropical Medicine, Antwerp. The first author is supported by a PhD scholarship from the Institute of Tropical Medicine, Antwerp under the DGD grant mentioned above.
